# Comparative Release of Platelet-Derived Growth Factor-AA and Evaluation of Osteoblastic Proliferation of Two Liquid Platelet-Rich Fibrin Formulations (C-PRF and I-PRF): An In Vitro Study

**DOI:** 10.1155/ijbm/3568968

**Published:** 2025-03-18

**Authors:** Nithyakalyani Ramesh, Jayanthi Anbalagan, Muthukumar Santhanakrishnan, Alan M. Punnoose, Rajalakshmi Shanmugham, John Kirubaharan

**Affiliations:** ^1^Department of Periodontics, Sri Ramachandra Institute of Higher Education and Research, Chennai, India; ^2^Centre for Regenerative Medicine and Stem Cell Research, Faculty of Clinical Research, Sri Ramachandra Institute of Higher Education and Research, Chennai, India; ^3^Department of Veterinary Microbiology, Madras Veterinary College, Chennai, India

**Keywords:** bone regeneration, concentrated platelet-rich fibrin, injectable platelet-rich fibrin, xenografts

## Abstract

**Context:** Several biomaterials have been developed in the field of tissue regeneration, in addition to creating a “foreign body reaction,” they lack the cellular components that are necessary for the regeneration process and, therefore, do not fulfill their purpose satisfactorily. In this regard, the use of platelet concentrates has gained popularity. However, sufficient scientific evidence is still lacking for the use of platelet concentrates, especially platelet-rich liquid fibrin preparations in combination with xenografts. The results of the present study would give an indication of the advantages of using the combination of xenogenic bone graft in combination with liquid formulations of platelet concentrates in regenerative periodontal therapy.

**Aims:** This in vitro study was performed to compare and evaluate the differential release profile and osteogenic potential of liquid formulations of platelet concentrates, namely, concentrated platelet-rich fibrin (C-PRF) and injectable platelet-rich fibrin (I-PRF).

**Methods and Materials:** The differential release profile of platelet-derived growth factor-AA (PDGF-AA) and osteogenic potential of liquid formulations of platelet concentrates was evaluated using samples collected from four periodontally healthy female volunteers by ELISA and alkaline phosphatase (ALP) assay with the help of human osteosarcoma cell lines (Saos-2).

**Statistical Analysis:** Statistical analysis of growth factor release profile and estimation of ALP activity was performed using the Kruskal–Wallis test to compare the mean difference between the following groups: C-PRF and I-PRF with and without bone graft. Data were analyzed using SPSS Version 21 software.

**Results and Conclusions:** This study clearly shows the advantage of using liquid platelet concentrates in combination with bone grafts compared with bone grafts alone. The study further suggested that the use of C-PRF could be beneficial in regenerative periodontal therapy.

## 1. Introduction

Periodontal disease states often result in the loss of supporting tooth tissues, leading to the gradual destruction of periodontal ligament and alveolar bone, justifying the need for regenerative periodontal therapy [1]. The goal of regenerative periodontal therapy is to completely restore the tooth's supporting apparatus that has been lost due to inflammatory periodontal disease or injury [2]. Regeneration of the periodontium has remained an elusive goal because, in addition to creating a “foreign body reaction,” most biomaterials developed lack the cellular components necessary for regeneration to occur and, therefore, do not satisfactorily serve the purpose.

In this scenario, the use of platelet concentrates has gained popularity due to the fact that platelet-rich fibrin (PRF) is a self-supporting matrix with the presence of several growth factors, cytokines, and leukocytes that create an efficient network for cell migration with increased ability to promote angiogenesis and tissue ingrowth [3].

Choukron et al. in 2001, introduced PRF, a second-generation platelet concentrate. PRF accumulates platelets and releases cytokines in the fibrin clot, resulting in slow, sustained release through the natural maturation and reorganization of the clot [4]. Platelets rich in fibrin is often used to enhance soft tissue wound healing and fight bacterial sepsis through the presence of leukocytes within it [5]. Despite the various advantages of PRF, one of the most prominent limitations of its use is the rapid breakdown of the fibrin network. The rapid rate of disintegration fails to fully exploit its potential as a regenerative material, especially during the initial events of wound healing [6]. In this regard, the use of PRF in combination with xenografts has proven advantageous, as xenografts have a slow substitution rate leading to delayed resorption of the graft material. However, there is a limited scientific basis to support the use of PRF, especially its solid forms, in combination with xenografts, as the integrity of the fibrin network is compromised by the addition of grafts, which could be detrimental to the sustained release of growth factors from platelet concentrates.

Over the years, the potential of liquid platelet concentrates has gained importance. Although platelet-rich plasma provides more rapid delivery of growth factors to the target site as compared with PRF and CGF the transmission of infectious diseases and coagulopathies serves as a crucial limitation to the PRP technique as it involves the use of bovine thrombin to initiate the clotting mechanism. In contrast to PRP, PRF and CGF require only centrifuged autologous blood and, therefore, provide immunological biocompatibility [7]. In this regard, Miron and Ghanaati in 2017 found that a further reduction of the relative centrifugal force (RCF) to 60 G led to the introduction of an injectable PRF matrix (I-PRF) that did not require the use of anticoagulants [8]. The reduction of RCF led to an enrichment of I-PRF with a higher number of platelets and leukocytes found in I-PRF compared to other PRF-based solid matrices [9]. In addition, liquid formulations of PRF have the distinct advantage of being adsorbed to graft particles and because they lack a fibrin network, the release profile of growth factors would remain unaffected [9]. Furthermore, in 2019, Miron and colleagues discovered a new technique in which sequential pipetting of 1 mL layers of blood after centrifugation led to cell quantification in platelet concentrates. It was observed that in the original L-PRF protocols, a large number of cells accumulated in the buffy coat, which is present immediately above the red blood cell layer, with very few cells found in the upper PRF clot. This layer directly above the RBC layer has been defined as concentrated platelet-rich fibrin (C-PRF) [10].

Literature evidence assessing the efficacy of liquid formulations of PRF in the release of growth factors remain limited. In addition, although platelet concentrates have been used extensively in periodontal regeneration, their efficacy in bone regeneration has not been conclusive.

In this regard, this in vitro study was conducted to compare and evaluate the differential release profile of growth factor PDGF-AA and osteogenic potential of liquid formulations of platelet concentrates, namely, C-PRF and I-PRF.

## 2. Materials and Methods

All materials and procedures were approved by the Institutional Ethics and Review Board, Sri Ramachandra Institute of Higher Education and Research (REF: CSP/19/SEP/80/304) prior to initiation of the study.

### 2.1. Sample Selection

The present in vitro study included 4 systemically and periodontally healthy female volunteers aged 20–25 years who were nonsmokers and did not take any medications. A complete blood count of the participants was also examined before starting the experiments to confirm the standard cell count ranges (1.5–4.5 lakhs/cu.mm). Platelet counts were taken before sampling for the study and compared between individuals to avoid variation due to platelet counts at baseline and after the release profile (Table 1).

Patients with systemic diseases, smokers, patients who consumed alcohol, pregnant and lactating women, those who have undergone periodontal treatment, and those who have consumed antibiotics in the last 3 months were not selected for this study. Informed consent was obtained from all participants.

**Figure 1:**
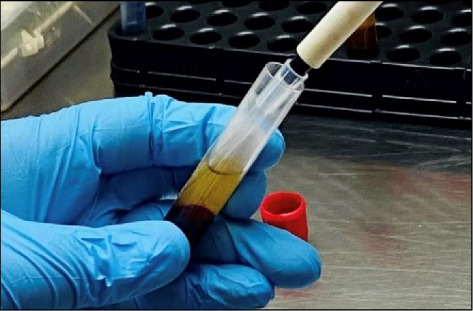
Procurement of C-PRF.

PRF concentrates were prepared as previously described by Sam, Vadakkekuttical, and Amol [6]. Blood samples were collected by venipuncture from the forearm in the antecubital vein into a 10 mL sterile glass vacuum tube for C-PRF preparations and into a 10 mL plastic tube for I-PRF preparation (VACUTECH, INDIA). Blood samples were immediately centrifuged using a table centrifuge (EPPENDORF, 5804 R) at 60 G for 3 minutes at room temperature to prepare I-PRF. To prepare C-PRF, samples were centrifuged using the L-PRF protocols at 700 G for 3 minutes at room temperature, after which 1 mL of the upper fluid layer directly above the RBC layer was collected as C-PRF (Figure 1).

### 2.2. Growth Factor Release Profile [11]

Harvested PRF samples were placed in tissue culture 6-well plates containing McCoys growth medium and incubated at 37°C with 5% CO2 to allow growth factor release. The amount of released growth factor PDGF-AA was determined using a commercial ELISA kit (E-EL-H1575, Elabscience Biotechnology Co.Ltd, China) according to the manufacturer's protocols at periodic intervals of 15 min, 60 min, 8 h, 1 day, 3 days, and 10 days. Approximately 1 mL of culture medium was removed from each of the incubated wells at each of these time points and supplemented with fresh medium. The collected samples were frozen at −20°C until further analysis.

**Figure 2:**
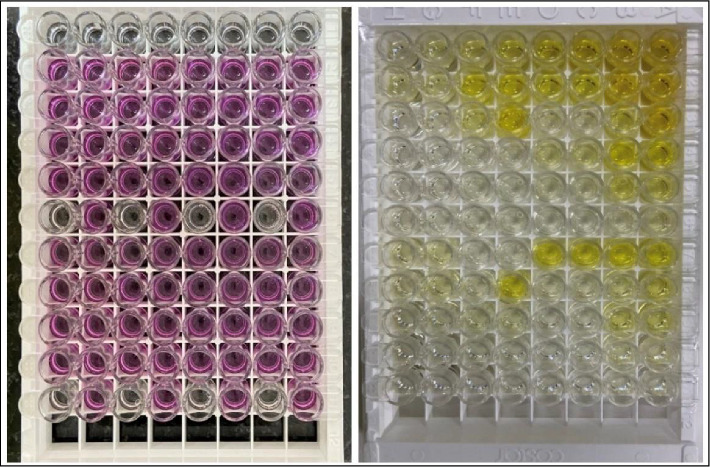
Samples loaded onto the ELISA plate for measuring the levels of PDGF-AA.

At the desired time points, PDGFAA (DY221, range = 15.63–1000 pg/mL) was quantified by ELISA. A total of 100 μL of assay diluents together with 100 μL of sample were incubated for 1.5 h at 37°C in antibody-precoated 96-well plates (Figure 2).

**Figure 3:**
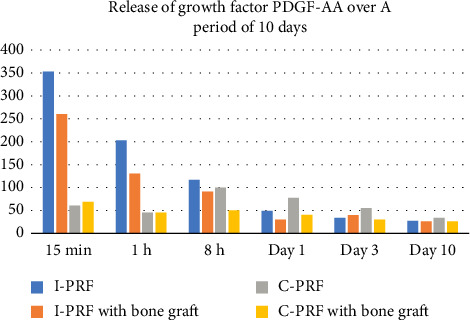
Release of growth factor PDGF-AA over a period of 10 days.

After removing the liquid, 100 μL of biotinylated antibody was added to the wells and incubated at 37°C for 1 h. Wash buffer was used to wash the wells three times and 100 μL of HRP conjugate was added for incubation for 30 min at 37°C. After washing the wells five times, 90 μL of substrate reagent was added for 15 min. The absorbance was measured at 450 nm immediately after the addition of 50 μL stop solution (Figure 2). The obtained results were compiled into a table (Figure 3 and Table 2).

### 2.3. Osteogenic Potential of C-PRF and I-PRF [12]

Human osteosarcoma cell lines (Saos-2) were obtained from the National Cell Science Center, NCCS, Pune, India, and were maintained in Mc Coys medium (Sigma) and 15% fetal bovine serum (FBS; Gibco). The medium was supplemented with antibiotics (50 μg/mL penicillin and 60 μg/mL streptomycin) and L-glutamine (4 mM final concentration), then the cells were incubated at 37°C in 5% CO2. A routine cell-seeding density of 2 × 105 cells per T25 flask was performed. A total of 60%–70% confluence was obtained within 3–5 days, after which the cells were ready for passaging. Cells were then detached from the substrate with IX Trypsin-EDTA (0.1% each in PBS for 2–3 min at 37°C) after a brief rinse in PBS (without Ca and Mg) to remove traces of FBS containing trypsin inhibitors and trypsin quenching with medium containing 10% serum (GM) and the medium was changed every 2 days. Harvesting/splitting of stock cultures was done at 70%–80% confluence.

### 2.4. Alkaline Pphosphatase (ALP) Activity

ALP activity was estimated using a commercial kit (CCK035, HiMedia Laboratories, Bengaluru, India) as per manufacturer's instructions [13]. Cells were cultured in growth media and after 2 days of seeding, and the wells were supplemented with 150 μL of PRF supplemented with or without 100 mg of xenograft (Geistlich *Bio-Oss)* into the growth media. The spent media was changed every alternate day and induction was carried out for 10 days. ALP activity was measured from the tissue culture supernatant from the identified tissue culture wells at specific period—namely, Day 3, Day 7, and Day 10.

About 167 mM solution of ρ-nitrophenyl phosphate was dissolved in 248 mg ALP substrate in 1 mL of cell culture grade water. The solution was stored in amber colored bottle at 2°C–8°C until used. Appropriate quantities of assay buffer and substrate solution were added in blank, control, and sample tubes. The tubes were equilibrated at 37 degrees Celsius by keeping in a water bath. A total of 20 μL of ALP control solution was added to the control tube and 20 μL of the test sample was added to test tube. The contents were immediately mixed by inversion and the increase in absorbance at 405 nm upto 5 min was recorded.

The concentration of ALP was calculated using the following formula:

(1) $$\begin{array}{c} \text{ALP }\left(\text{units}/\text{mL}\right)=\frac{\left(\Delta A405\text{ nm}/\text{min Test}-\Delta A405\text{ nm}/\text{min Blank}\right)\times \text{DF}\times \text{TV}}{18.5\times \text{EV}}, \end{array}$$

where DF = dilution factor, TV = total volume of the assay (mL), EV = volume of the enzyme/sample used for assay (mL), and 18.5 = millimolar extinction coefficient of ρNPP at 405 nm.

**Figure 4:**
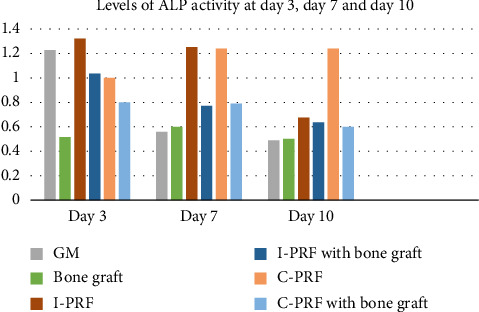
Levels of ALP activity at Day 3, Day 7, and Day 10.

The results obtained were tabulated (Tables 3 and 4 and Figure 4).

### 2.5. Statistical Analysis

Statistical analysis of growth factor release profile and estimation of ALP activity was performed using the Kruskal–Wallis test to compare the mean difference between the following groups: C-PRF and I-PRF with and without bone graft. Data were analyzed using SPSS software Version 21(Tables 2 and 4).

## 3. Results

### 3.1. Growth Factor Release Profile

The release of PDGF-AA growth factor was analyzed using the Enzyme-Linked Immunosorbent Assay. At early time points, after 15 min, 1 h, and 8 h; higher levels of PDGF-AA were released from I-PRF (353.6 ± 13.01 pg/mL, 202.3 ± 212.89 pg/mL, and 115.6 ± 105.29 pg/mL) when compared with C-PRF (60.89 ± 18.08 pg/mL, 45.5 ± 30.34 pg/mL, and 100.6 ± 28.71 pg/mL). However, at time points of Day 1, Day 3, and Day 10, a marginal increase in the levels of PDGF-AA were released from C-PRF (76.71 ± 12.86 pg/mL, 55.3 ± 16.03 pg/mL, and 33.35 ± 14.02 pg/mL) when compared with I-PRF (48.08 ± 2.42 pg/mL, 33.21 ± 18.58 pg/mL, and 28.64 ± 31.79 pg/mL). It was also found that the release of PDGF-AA in the combination of C-PRF and I-PRF with bone graft group was lesser in comparison with the only PRF groups at all estimated time points except at 15 min and 1 h, where a marginal increase in levels of PDGF-AA was observed in C-PRF with bone graft group (69.70 ± 41.7 pg/mL and 45.81 ± 20.35 pg/mL) in comparison with C-PRF (60.89 ± 18.08 pg/mL and 41.53 ± 30.34 pg/mL) (Figure 3 and Table 2). The release of PDGF-AA from the only bone graft group at 15 min was found to be negligible (6.28 pg/mL) (Table 2) in comparison with the release profile of both C-PRF and I-PRF in combination with bone grafts (69.7 ± 41.75 pg/mL and 260.1 ± 216.6 pg/mL). Intergroup comparison of the platelet concentrates (C-PRF and I-PRF and C-PRF and I-PRF with bone graft) did not show any statistically significant difference at different time points (Table 2).

### 3.2. ALP Activity

The ALP activity was measured for the designated groups at time points Day 3, Day 7, and Day 10. It was observed that at Day 3, there was an initial increase in the levels of ALP activity in the I-PRF group (1.324 ± 0.466 units/mL) in comparison with C-PRF (1.002 ± 0.477 units/mL) (Figure 4). However, at Day 7, the release of ALP was found to be comparable between C-PRF and I-PRF (Figure 4). It was further observed that at Day 10, the levels of ALP activity were higher in the C-PRF groups (1.241 ± 0.590 units/mL) over the I-PRF groups (0.679 ± 0.366 units/mL) (Figure 4). In both the C-PRF and I-PRF groups, it was found that the combination of xenografts yielded lesser ALP activity in comparison with the only PRF groups; however, their ALP activity was more pronounced in comparison with the only xenografts group (0.515 units/mL at Day 3, 0.603 units/mL at Day 7, and 0.505 units/mL at Day 10) (Table 3). Intergroup comparison of the platelet concentrates (C-PRF and I-PRF and C-PRF and I-PRF with bone graft) did not show any statistically significant difference at different time points (Table 4). However, the trend showed an increase in ALP activity in the I-PRF group at earlier time points and in the C-PRF group at later time points.

## 4. Discussion

Over the years, there has been an exponential demand for a biomaterial that can satisfactorily serve the purpose of regenerative periodontal therapy. Although several materials have been introduced, certain important concerns regarding their use, such as the lack of cells and signaling molecules, have necessitated the need to consider a more natural source of biomaterial.

In this regard, the use of platelet concentrates, which are the autologous source of the tissue engineering construct, has proven to be a viable option. Platelet concentrates constitutively express scaffolds in the form of fibrin, cells in the form of leukocytes, and signaling molecules in the form of growth factors. Several generations of platelet concentrates have been introduced in the past. From the introduction of platelet-rich plasma, which requires an anticoagulant, to solid forms of PRF (A-PRF, L-PRF, and T-PRF) and, more recently, liquid formulations of PRF, the use of platelet concentrates has revolutionized periodontal therapy over the years.

Despite the various advantages that platelet concentrates offer, one of the main disadvantages is their rapid rate of disintegration. In this regard, the use of PRF in combination with graft particles has become increasingly important. Among the various graft materials available, allografts are one of the most sought-after options due to their osteoinductive potential. However, after demineralization, not only is their osteoinductive potential compromised but also their structural integrity is lost, which leads to faster resorption of the graft particles [14]. In addition, other graft materials such as autografts result in limited material, donor site morbidity, unpredictable bone quality, and postoperative discomfort, and alloplasts show faster resorption, especially within 8 weeks [14]. Currently used biologics such as derivatives of the tooth enamel matrix, commercially available as EMDOGAIN, although it has shown promise as a regenerative material, it has the disadvantage of insufficient economic viability, which limits its use in routine periodontal practice [15]. In this scenario, the use of xenograft in combination with PRF has been shown to be useful because xenografts have a slow rate of substitution and, therefore, slow graft resorption. However, there is a lack of scientific evidence supporting the use of this combination. The amount of evidence describing the effect of platelet-rich liquid fibrin combined with xenografts on bone regeneration and growth factor release profile is scarce.

In this scenario, the present in vitro study was conducted to compare and evaluate the differential release profile of growth factor PDGF-AA and osteogenic potential of liquid formulations of platelet concentrates, namely, C-PRF and I-PRF with and without a combination of xenogeneic bone substitute (Geistlich Bio-Oss).

This study included four systemically and periodontally healthy female volunteers aged 20–25 years. The release profile and osteogenic potential between liquid formulations of C-PRF and I-PRF with and without a combination of xenogeneic bone substitute (Geistlich Bio-Oss) were compared. A xenogeneic bone substitute was chosen as the graft because of its advantages of being biocompatible and structurally similar to human bone, but more importantly, because of the slow rate of substitution that would compensate for the rapid breakdown of PRF [16].

This study checked the release profile of PDGF-AA from C-PRF and I-PRF samples. PDGF-AA has been shown to be an important factor in promoting the regeneration of bone, periodontal ligament and cementum [17]. Furthermore, among the four PDGF isomers (AA, AB, BB, CC, DD, and AB), PDGF-AA was found to be associated with active fibroblasts, extracellular matrix deposition, and the ability to achieve neovessel formation to a greater extent compared with other PDGF isomers and thus PDGF-AA qualified as a suitable marker for this study. A total of 100 mg of xenogeneic bone substitute was incubated with 150 μL of I-PRF or C-PRF, and the release profile and osteogenic potential of this combination was evaluated (Kyyak et al., 2020). The PDGF-AA release profile was quantified using an ELISA assay due to its distinct advantages over other immunoassay techniques, such as its high specificity and sensitivity due to the antigen–antibody reaction and a high degree of accuracy [18]. The assay was performed at time points 15 min, 60 min, 8 h, Day 1, Day 3, and Day 10. The following time points were taken into account because they correspond to the early and late stages of inflammation in the periodontal wound healing process [19]. Following the initiation of the wound healing process, after 15 min, there is accumulation of red blood cells that occurs almost immediately. Within 1–8 h, there is a predominant accumulation of neutrophils and monocytes, indicating the early phase of inflammation. Within 1–3 days, the late inflammatory phase dominates the healing period as macrophages migrate into the wound, granulation tissue formation follows, and by 7–10 days cell-rich connective tissue attachment may begin to form [17]. Therefore, the abovementioned time points were chosen for their clinical relevance.

The results of the present study showed that at early time points after 15 min, 1 h, and 8 h, higher levels of PDGF-AA were released from I-PRF (353.6 ± 13.01 pg/mL, 202.3 ± 212.89 pg/mL, and 115.6 ± 105.29 pg/mL) when compared with C-PRF (60.89 ± 18.08 pg/mL, 45.5 ± 30.34 pg/mL, and 100.6 ± 28.71 pg/mL) (Table 2). However, at time points of Day 1, Day 3, and Day 10, a marginal increase in the levels of growth factor release from C-PRF (76.71 ± 12.86 pg/mL, 55.3 ± 16.03 pg/mL, and 33.35 ± 14.02 pg/mL) was observed when compared with I-PRF (48.08 ± 2.42 pg/mL, 33.21 ± 18.58 pg/mL, and 28.64 ± 31.79 pg.mL) (Table 2). The elevated release of growth factors from I-PRF at early time points could be attributed to the fact that at low centrifugation forces (60G force for I-PRF as compared with 700 G force for C-PRF), a higher percentage of cells including platelets and leukocytes remain in the upper phase of centrifugation tubes, where I-PRF is collected, thereby providing more cells capable of assisting in tissue regeneration and the release of prowound healing molecules [9]. The rise in growth factor release from I-PRF at early time points is of clinical relevance as it contributes to the early phase of wound healing. A similar trend was observed by Miron et al. in 2017 while comparing I-PRF with PRP wherein although the growth factor release from I-PRF was high at early time points (15 min, 1 h, and 8 h), a decline was observed at the remaining time intervals [20]. The marginal increase in the levels of growth factor release from C-PRF in comparison to I-PRF at later time points could probably be due to the sustained release of growth factors from C-PRF over a period of time. The rise in growth factor release from C-PRF at later time points is of clinical relevance as the later time points marks the beginning of macrophage migration and the formation of a cell-rich connective tissue attachment [19].

The present study did not observe a consistently elevated level of growth factor release from C-PRF as opposed to Miron et al. in 2020, who demonstrated that C-PRF had a more consistent release of growth factors in comparison with I-PRF over a period of time [21]. The probable reason for this could be that in the present study, C-PRF was prepared with the use of a fixed angle centrifuge, in contrast to a horizontal centrifuge used by Miron et al. who reported in 2019 that the use of a horizontal centrifuge is more beneficial owing to its uniform layer separation, making the procurement of the concentrated layer of PRF more accurate and precise [10].

In this study, the release profile of PDGF-AA from the single xenograft group at 15 min was observed to be negligible (6.28 pg/mL) (Table 2). However, the addition of platelet concentrates to xenografts was shown to be beneficial, as a distinct increase in release potential was observed. A smaller release profile was observed in the PRF bone graft group time points compared with the PRF alone group, which could be due to a possible masking effect of the graft material, thereby hindering the release potential from the PRF. However, it was observed that the potential of the combination of C-PRF and bone graft was increased compared with the only C-PRF group at 15 min and 1 h. It could be hypothesized that since the release profile of C-PRF appears to be delayed, the addition of bone graft is particularly beneficial at early time points as evidenced by the increased growth factor release. In addition, it was observed that the potential of the combination of PRF and bone graft was increased compared with the bone graft alone group time points, clearly demonstrating the beneficial effect of the combination of platelet concentrates with bone grafts. The release pattern of C-PRF and I-PRF with xenografts could not be compared with other studies because, to our knowledge, this is the first study to investigate the growth factor release profile from a combination of PRF liquid formulations and xenografts.

ALP expression was evaluated to assess the osteogenic potential of C-PRF and I-PRF liquid preparations as ALP is a membrane-bound enzyme released from polymorphonuclear neutrophils during inflammation and periodontal ligament osteoblasts and fibroblasts during periodontal regeneration [22]. The estimation of ALP levels will, therefore, provide a clear indication of the osteogenic and regenerative potential of the biomaterial in question. Human osteosarcoma (Saos-2) cell lines were used to mimic human osteoblasts because this cell line has several osteoblastic features and could be used as a source of bone-related molecules [23]. Furthermore, Saos-2 cells can be fully differentiated in a manner that osteoblastic cells naturally do [23].

The ALP activity was measured for the designated groups at time points Day 3, Day 7, and Day 10. It was observed that at Day 3, there was an initial increase in the levels of ALP activity in the I-PRF group (1.32 ± 0.466 units/mL) in comparison with C-PRF (1.002 ± 0.477 units/mL) (Table 4). However, at Day 7, the release of ALP was found to be comparable between C-PRF and I-PRF. It was further observed that at Day 10, the levels of ALP activity were higher in the C-PRF groups (1.241 ± 0.590 units/mL) over the I-PRF groups (0.679 ± 0.366 units/mL) (Table 4). A probable reason for the increased cellular activity of I-PRF at early time points could be the additional incorporation of leukocytes as well as the presence of fibrin proteins that are yet to coagulate. The results observed were in agreement with the study done by Kyyak et al. in 2020, who observed that an elevated level of ALP expression that was seen at Day 3 and Day 7 in the xenograft with the I-PRF group; however, the levels of ALP activity declined at Day 10. The elevated levels of ALP activity in the C-PRF group at Day 10 could be due to the sustained release potential of platelets and leukocytes, which in turn might have an effect on cellular activity. The increase in ALP activity at Day 10 is of clinical significance because it may correlate with the migration of undifferentiated mesenchymal stem cells, which further leads to the proliferation of osteoprogenitor cells [22].

In the present study, in the only xenografts group, the levels of ALP activity were found to be 0.515 units/mL at Day 3, 0.603 units/mL at Day 7, and 0.505 units/mL at Day 10 (Table 3). However, in both the C-PRF and I-PRF groups, there was a substantial increase in the levels of ALP activity when added to bone grafts. It was found that although the combination of xenografts with PRF yielded lesser ALP activity in comparison with the only PRF groups, the osteogenic potential of PRF and bone grafts when used in combination was more pronounced when compared with the only bone grafts group. It is safe to assume that a probable reason for this is that PRF can improve the properties of graft materials by providing cytokines, platelets, leukocytes, and circulating stem cells. The results of the present study were in agreement with the results obtained from clinical trials conducted by Lekovic et al. in 2012, Xuan et al. in 2014, and Ezirganli et al. in 2015, who all reported that the use of PRF in combination with xenografts is more beneficial than the use of only xenografts in the management of intrabony defects, sinus lift procedures, and guided bone regeneration. More recently, a systematic review by Vini et al. in 2023 revealed that PRP used as an adjunct with a xenograft resulted in a significant reduction in pocket depth and greater clinical attachment level gain in comparison with xenograft alone [23]. All the authors unanimously suggested that although PRF had slight potential in enhancing new bone formation as a result of early vascularization of bone tissues, they cannot be used alone and must be used in combination with bone grafting materials [24].

This study had the following limitations. The release profile of more than one growth factor (PDGF-AA) could have been evaluated and the effects of platelet concentrates on osteoblast differentiation would have provided a more definitive indication of the mineralization potential of the biomaterial. In addition, the use of primary cell culture may have provided more definitive results.

## 5. Conclusion

Within the limitations of this study, the following conclusions can be drawn:

• This study clearly demonstrated the benefit of using platelet concentrates in combination with bone grafts, as evidenced by increased levels of PDGF-AA release and increased ALP activity compared with bone grafts alone.
• The differential release profile of PDGF-AA and osteogenic potential of C-PRF is greater than that of I-PRF at later time points, suggesting that the use of C-PRF could be beneficial in regenerative periodontal therapy.

Future studies evaluating the in vivo efficacy of C-PRF in regenerative periodontal therapy are required to arrive at a definitive conclusion about the inherent potential of C-PRF as a viable approach for periodontal regeneration.

## Figures and Tables

**Table 1:** Baseline platelet count.

Sample	Baseline platelet value (whole blood) (lakhs/cumm)
Patient 1	2.92
Patient 2	2.83
Patient 3	2.94
Patient 4	2.87

**Table 2:** Differential release profile of PDGF-AA measured in pg/mL.

	Groups	Mean	Std. deviation	*p* value^∗^
Fifteen minutes	I-PRF	353.6	13.01076	0.149
	I-PRF with BG	260.1	216.63630	
	C-PRF	60.89	18.08072	
	C-PRF with BG	69.7	41.75466	

Sixty minutes	I-PRF	202.3	212.89571	0.539
	I-PRF with BG	130.5	99.34143	
	C-PRF	41.5	30.34549	
	C-PRF with BG	45.8	20.35053	

Eight hours	I-PRF	115.6	105.29527	0.761
	I-PRF with BG	90.9	75.51900	
	C-PRF	100.6	28.71561	
	C-PRF with BG	50.89	45.04270	

Day one	I-PRF	48.08	2.42538	0.198
	I-PRF with BG	30.09	5.69221	
	C-PRF	76.71	12.86934	
	C-PRF with BG	39.4	35.25634	

Day three	I-PRF	33.21	18.58984	0.446
	I-PRF with BG	41.09	2.89914	
	C-PRF	55.32	16.03011	
	C-PRF with BG	30.6	19.43837	

Day ten	I-PRF	28.64	31.79859	0.983
	I-PRF with BG	27.43	23.99920	
	C-PRF	33.35	14.02193	
	C-PRF with BG	25.91	15.05430	

*Note:* Release of PDGF-AA from bone grafts alone at 15 min—6.28Pg/mL.

Abbreviations: BG, bone graft; C-PRF, concentrated platelet-rich fibrin; I-PRF, injectable platelet-rich fibrin.

⁣^∗^*p* value < 0.05 was considered significant using the Kruskal–Wallis test.

**Table 3:** ALP release measured in units/mL for growth media and bone graft.

	Day 3	Day 7	Day 10
Growth media	1.23	0.563	0.491
Bone graft	0.515	0.603	0.505

**Table 4:** Intergroup comparison of ALP activity over a 10-day period measured in units/mL.

	Groups	Mean	Std. deviation	*p* value
Day three	I-PRF	1.324	0.466978	0.571
	I-PRF with BG	1.037	0.572776	
	C-PRF	1.002	0.477177	
	C-PRF with BG	0.802	0.404523	

Day seven	I-PRF	1.254	0.442336	0.344
	I-PRF with BG	0.772	0.215305	
	C-PRF	1.246	0.434829	
	C-PRF with BG	0.793	0.467370	

Day ten	I-PRF	0.679	0.366186	0.355
	I-PRF with BG	0.643	0.284896	
	C-PRF	1.241	0.590909	
	C-PRF with BG	0.606	0.446735	

Abbreviations: BG, bone graft; C-PRF, concentrated platelet-rich fibrin; I-PRF, injectable platelet-rich fibrin.

⁣^∗^*p* value < 0.05 was considered significant using the Kruskal–Wallis test.

## Data Availability

The data used to support the findings of this study are available on request from the corresponding author.
