# Correction to “Comparative Release of Platelet-Derived Growth Factor-AA and Evaluation of Osteoblastic Proliferation of Two Liquid Platelet-Rich Fibrin Formulations (C-PRF and I-PRF): An In Vitro Study”

**DOI:** 10.1155/ijbm/9852454

**Published:** 2025-09-23

N. Ramesh, J. Anbalagan, M. Santhanakrishnan, A. M. Punnoose, R. Shanmugham, and J. Kirubaharan, “Comparative Release of Platelet-Derived Growth Factor-AA and Evaluation of Osteoblastic Proliferation of Two Liquid Platelet-Rich Fibrin Formulations (C-PRF and I-PRF): An In Vitro Study,” *International Journal of Biomaterials* 2025 (2025): 3568968, https://doi.org/10.1155/ijbm/3568968.

In the article titled “Comparative Release of Platelet-Derived Growth Factor-AA and Evaluation of Osteoblastic Proliferation of Two Liquid Platelet-Rich Fibrin Formulations (C-PRF and I-PRF): An In Vitro Study,” there was an error in affiliation number 2. In the corrected affiliation “Centre for Regenerative Medicine and Stem Cell Research” has been changed to “Stem Cell and Regenerative Biology Laboratory”. The corrected affiliation appears below:

Stem Cell and Regenerative Biology Laboratory, Faculty of Clinical Research, Sri Ramachandra Institute of Higher Education and Research, Chennai, India

We apologize for this error.

